# Real-World Utilization of Daratumumab in Front-Line Treatment of Newly Diagnosed Multiple Myeloma Across a Regional Academic–Community Network

**DOI:** 10.3390/cancers18121899

**Published:** 2026-06-11

**Authors:** Poy Theprungsirikul, Amer Al-Musa, Noffar Bar, Sabrina L. Browning, Terri Parker, Natalia Neparidze

**Affiliations:** 1 Department of Internal Medicine, Section of Hematology and Medical Oncology, Yale School of Medicine, New Haven, CT 06510, USA; poy_theprungsirikul@dfci.harvard.edu (P.T.); amer.al-musa@yale.edu (A.A.-M.); noffar.bar@yale.edu (N.B.); sabrina.browning@yale.edu (S.L.B.); terri.parker@yale.edu (T.P.); 2 Dana Farber Cancer Institute, Boston, MA 02215, USA

**Keywords:** myeloma, newly diagnosed, daratumumab induction

## Abstract

Background: The addition of CD-38 monoclonal antibody daratumumab (DARA) to front-line therapy for newly diagnosed multiple myeloma (NDMM) has improved outcomes. The real-world (RW) adoption of treatments remains inconsistent. We aimed to evaluate the adoption of DARA regimens in the treatment of NDMM. Methods: We conducted a retrospective study at a multi-site academic–community healthcare system from August 2020–September 2023. Patients (n = 414) were ≥18 years with NDMM upon initiating front-line therapy. Results: The median age was 69; most were male (60.6%, n = 251), white (69.1%, n = 286), and non-Hispanic (89.6%, n = 371). At diagnosis, 52.2% (n = 216) had R-ISS stage II, and the performance status was preserved, with 69.3% (n = 287) having ECOG 0–1. The adoption of DARA-based induction was incomplete (53.9%, n = 223) and was higher at the academic myeloma center than the community sites (63.9% vs. 47.7%; *p* = 0.001). At three months after treatment initiation, patients receiving DARA-based induction achieved deeper responses than those treated without DARA. The distribution of IMWG responses favored the DARA group, with a higher proportion attaining VGPR or better (65.0% [n = 145] vs. 51.3% [n = 98]). Conclusions: DARA-based regimens for NDMM were underutilized and notably less common at community sites. Coordinated implementation strategies at the provider and community levels may narrow adoption gaps and translate trial-proven benefits into routine practice.

## Simple Summary

The incorporation of CD38 monoclonal antibodies (mAb) in the front-line treatment of newly diagnosed multiple myeloma (MM) has transformed the therapeutic landscape and resulted in improved depth of responses, minimal residual disease (MRD)-negative responses, progression-free survival, and improved overall survival outlook in myeloma. While the initial landmark studies describing daratumumab (DARA)-based quadruplet induction regimens reported the encouraging results in early 2020, the universal adoption and the incorporation of CD38mAbs in the front-line care of MM in general practice have been delayed. In this retrospective study, we examined the adoption of daratumumab-based front-line regimens for newly diagnosed multiple myeloma. We observed that DARA-based regimens were underutilized and notably less common at community sites compared with the main academic site where the care is sub-specialized based on the disease. Front-line DARA use was more common among patients with a better Eastern Collaborative Oncology Group (ECOG) performance status of 0–1. Importantly, patients who received DARA-based induction achieved deeper responses than those treated without DARA. A higher proportion of patients receiving DARA-based induction attained a very good partial response (VGPR) or better. Dissemination of the groundbreaking research information and coordinated implementation strategies at the provider and community levels may narrow adoption gaps and translate trial-proven benefits into routine practice.

## 1. Introduction

Treatment of multiple myeloma (MM), a plasma cell neoplasm, has undergone significant therapeutic advancements over the past two decades. Three-drug combination regimens containing novel agents such as immunomodulatory agents (IMiDs) and proteasome inhibitors (PIs) used to be the established standard regimens for induction therapy for almost two decades, as they resulted in deep responses, prolonged duration of remission, and improved overall survival (OS). A combination of bortezomib (velcade), lenalidomide (revlimid), and dexamethasone (VRd) was the standard induction regimen in the United States from 2008 to 2020. VRd showed high efficacy rates in initial published reports from phase II studies [1]. Subsequently, VRd was incorporated as the standard front-line therapy in the myeloma guidelines and represented the backbone treatment in a large international phase III trial (IFM/DFCI) [2,3]. This regimen demonstrated deep responses, with a very good partial response rate of over 70% and complete response rates of 40%. Further data on the VRd regimen came from the phase II EVOLUTION and phase III SWOG S0777 trials, again revealing encouraging response rates for VRD [4,5].

The introduction of monoclonal antibodies, particularly daratumumab (DARA), has revolutionized treatment paradigms in both newly diagnosed multiple myeloma (NDMM) and relapsed/refractory multiple myeloma (RRMM). DARA, the first-in-class CD38-targeted fully human monoclonal antibody, has played an instrumental role in the improved management of patients with MM. DARA exerts both direct and indirect antitumor activity, with diverse mechanisms of action. These therapeutic modalities include direct induction of apoptosis as well as several classic FC-dependent immune-mediated effects through complement-dependent cytotoxicity (CDC), antibody-dependent cellular cytotoxicity (ADCC), and antibody-dependent cellular phagocytosis (ADCP) [6]. The different immunomodulatory functions of daratumumab have been well documented and are thought to play a critical role in daratumumab’s anti-plasma cell activity. Daratumumab has been successfully used in clinical practice for RRMM since 2015 based on initial trials leading to its FDA approval [7,8]. Several landmark clinical trials proved this agent to be safe and efficacious in combination with other anti-plasma cell therapies for the treatment of RRMM. These important studies included the POLLUX trial, where daratumumab plus lenalidomide and dexamethasone (DRd) significantly reduced the risk of progression or death compared to lenalidomide and dexamethasone alone, and the CASTOR trial, where daratumumab in combination with bortezomib and dexamethasone (DVd) demonstrated prolonged progression-free survival (PFS) and an improved overall response rate (ORR) compared to bortezomib and dexamethasone alone [9,10].

Subsequently, DARA showed promise in studies investigating its role in the induction or front-line setting [11]. Data on the safety and efficacy of daratumumab use in newly diagnosed MM (NDMM) were accumulated from the initial phase III randomized trials. The ALCYONE trial randomized newly diagnosed transplant-ineligible patients with CMM to receive VMP (velcade, melphalan, prednisone) with or without daratumumab [12]. The addition of daratumumab yielded a higher ORR and improved PFS. The MAIA study evaluated newly diagnosed transplant-ineligible MM patients treated with lenalidomide/dexamethasone with or without daratumumab. The addition of daratumumab resulted in deeper responses and superior PFS [13]. Additionally, a third phase III CASSIOPEIA trial treated transplant-eligible patients with newly diagnosed MM with VTD (velcade, thalidomide, dexamethasone) with or without daratumumab, followed by autologous stem cell transplant and consolidation. Here, the addition of daratumumab once again led to deeper responses and superior PFS. Fewer deaths were observed in the DARA–VTD arm, though the overall survival (OS) data were immature [14]. All three of the above trials demonstrated increased risk of grade 3 and 4 infections, neutropenia, lymphopenia, and infusion reactions with the addition of daratumumab.

DARA repeatedly demonstrated remarkable efficacy in combination with standard regimens, leading to deeper and more durable responses compared to traditional triplet therapies. Several landmark clinical trials have established the role of DARA in front-line treatments. The phase II GRIFFIN trial assessed DARA combined with lenalidomide, bortezomib, and dexamethasone (DARA–RVD) versus RVD alone in transplant-eligible patients with NDMM. The addition of DARA resulted in significantly higher rates of stringent complete responses and MRD-negative states following consolidation, with these advantages persisting over the course of extended follow-up. The subsequent analysis revealed durable PFS benefits and activity across all cytogenetic risk groups. The phase III PERSEUS trial unequivocally established DARA-based quadruplet therapy as a new standard of care for transplant-eligible patients with NDMM. In this large, randomized trial, subcutaneous DARA was added to the VRD induction and consolidation, followed by DARA–lenalidomide maintenance. With a median follow-up approaching four years, DARA–VRD reduced the risk of disease progression or death by 58% compared with VRD. This was accompanied by markedly higher rates of sustained MRD-negative and deep responses. In summary, the GRIFFIN and PERSEUS trials demonstrated that adding DARA to bortezomib, lenalidomide, and dexamethasone (D–VRD) significantly improved stringent complete response (sCR) rates, MRD negativity, and progression-free survival (PFS) in transplant-eligible (TE) NDMM patients compared to VRD alone [15,16,17]. Similar improvements in PFS and MRD negativity were observed by adding daratumumab to other triplet regimens incorporating thalidomide (T) and carfilzomib (K) in the CASSIOPEIA (D–VTD vs. VTD) and IFM 2018-04 (D–KRD vs. KRD) trials [18,19]. Similarly, in transplant-ineligible (TI) patients, the MAIA (D–RD vs. RD) and CEPHEUS (D–VRD vs. VRD) trials demonstrated improved PFS, OS, and MRD negativity with DARA-based regimens, without significantly increasing toxicity [20,21]. The addition of isatuximab (ISA) to the VRD has been proven to improve the efficacy, PFS and depth of responses among transplant-ineligible patients with MM [22]. The concurrent development of ISA-based quadruplet induction has provided an alternative CD38-oriented approach. The phase III GMMG-HD7 trial evaluated ISA combined with RVD (ISA–RVD) as induction therapy in transplant-eligible patients with NDMM. This combination demonstrated significantly higher MRD negativity rates following induction compared with RVD alone, establishing the biologic potency of ISA in this setting [23]. The updated analysis with longer follow-up confirmed that ISA–VRD induction translated into a statistically significant PFS benefit from the first randomization, regardless of the subsequent maintenance strategy, with MRD-negative responses continuing to deepen following the autologous stem cell transplantation [24].

A meta-analysis of seven randomized controlled trials involving both TE and TI NDMM patients showed that, compared to triplet regimens, quadruplet regimens incorporating an anti-CD38 mAb (daratumumab or isatuximab) not only improved the overall response rate (ORR; relative risk [RR], 1.03; 95% confidence interval [CI], 1.01–1.05) and PFS (hazard ratio [HR], 0.55; 95% CI, 0.46–0.66), but they also conferred an overall survival (OS) benefit (HR, 0.65; 95% CI, 0.53–0.79) [25].

While these trials have established mAb-based combinations as a new standard of care in NDMM, little is known about their real-world adoption. Historically, new oncology treatments have had variable uptake in clinical practice. For example, one study found that over 60% of eligible patients received anti-PD-1 agents within four months of their FDA approval, including for melanoma, non-small cell lung cancer, and renal cell carcinoma [26]. A large real-world analysis demonstrated that over 60% of eligible patients with non-small cell lung cancer do not receive appropriate biomarker-matched therapies, despite guidelines’ recommendations. Gaps in the adoption of this treatment approach occur at multiple steps, from testing to treatment selection, highlighting how operational inefficiencies and system fragmentation delay the adoption of novel therapies [27]. Studies have reported the inconsistent and variable adoption of DARA in front-line settings for MM [28,29]. Real-world data on the adoption of DARA-based regimens in the front-line induction of MM remain limited.

Several barriers have been cited to hinder the uptake of novel myeloma treatments. Clinicians may delay the adoption of newly studied regimens until they are endorsed by professional society and optimal positioning is clarified and added to the guidelines. Practice change is also not purely evidence driven, as established pathways are somewhat slow to alter. Academic–community gaps persist despite increasing collaboration and the adoption of electronic health records. Some academic centers prioritize clinical trial participation over off-protocol adoption. There is significant variability depending on practice setting and geography. Financial toxicity remains a major obstacle. The increasing costs of novel therapies pose significant financial barriers to timely and equitable treatment access in the United States, especially considering the increased use of costly combination regimens and prolonged treatment durations for MM in the maintenance and relapsed settings [30]. In addition, insurance-related out-of-pocket costs vary widely depending on insurance coverage. Medicare beneficiaries with low-income subsidies demonstrate higher utilization of immunomodulatory drugs than other Medicare recipients [30]. Lack of awareness of the guidelines’ recommendations among front-line practitioners as well as cancer patients and caregivers may also contribute. Health disparities further limit access, with racial and ethnic minorities, particularly African Americans, being less likely to receive appropriate treatment or undergo autologous stem cell transplantation [30]. Older patients—particularly those over 75—are at heightened risk of undertreatment despite functional heterogeneity. Geographic distance from treatment centers further exacerbates disparities [10]. Barriers may be different depending on the practice setting, with one study showing that financial constraints were perceived by referring oncologists as the primary barrier to implementing chimeric antigen receptor T-cell therapy (CAR-T) and T-cell engagers (TCEs) for myeloma, whereas logistical challenges were a greater concern for oncologists treating at referral centers [31]. Some daratumumab-specific barriers to implementation may include operational and safety considerations such as different administration and infusion strategies, infusion-related reactions, increased risk of infectious complications, and interference with blood transfusion management [32]. Since the publication of the GRIFFIN trial in 2020 (and later the PERSEUS trial), our institution has started utilizing DARA–RVD as the favored quadruplet regimen. Among triplet regimens, DARA–RD as per the MAIA trial is favored institutionally. In this study we examined the adoption of these DARA-based regimens in our institution compared to the older combinations such as VRD or RD. The analysis in our retrospective study is not focused on examining the efficacy of any specific quadruplet regimen, but rather the adoption of daratumumab in a front-line setting among all age groups with NDMM.

We hypothesized that despite its status as the new standard of care, DARA’s real-world adoption remains inconsistent, with financial toxicity, health disparities, and practice setting differences influencing its use as front-line therapy. In this study, we aimed to evaluate the real-world utilization of DARA in the front-line treatment of NDMM at the Yale Cancer Center Network, and to examine the factors influencing the use of DARA-based regimens. By assessing these trends, this study seeks to provide insights into the gaps between clinical trial evidence and real-world practice, ultimately guiding strategies to enhance the adoption of evidence-based therapies in NDMM management.

## 2. Materials and Methods

### 2.1. Study Design and Setting

We conducted a retrospective observational study within the Yale Cancer Center (YCC) Network, a multi-site academic–community system that includes a dedicated myeloma specialty center and affiliated community oncology practices. The study period spanned from 1 August 2020 to 30 September 2023 and coincided with the initial publication of landmark trials with quadruplet combinations and the increasing availability and insurance coverage of daratumumab (DARA)-containing front-line regimens in routine care. While stratification by triplet versus quadruplet regimens would provide additional clinical granularity, our analysis was designed to evaluate daratumumab adoption at a class level. Given the heterogeneity in regimen selection and patient characteristics, subgroup analyses would be subject to confounding and were beyond the scope of this retrospective study. Future analyses with larger cohorts and adjusted models will be undertaken as the next stage of this study.

### 2.2. Population

Eligible patients were adults (≥18 years) with a new diagnosis of multiple myeloma managed within the YCC Network who initiated systemic front-line therapy during the study window. Patients were identified through the electronic health record (EHR) using diagnosis codes and problem lists, augmented by the manual confirmation of clinical documentation. We used our institutional Joint Data Analytics Team (JDAT) to obtain the first cohort of patients with newly diagnosed MM. JDAT is a collaborative initiative that focuses on enhancing research analytics and reporting. It utilizes our institution’s customized data warehouse and various tools to support research activities across the institution. JDAT aims to facilitate data-driven decision-making and improve the efficiency of research processes by providing comprehensive data analysis and reporting services. Once the initial cohort of NDMM patients was obtained from JDAT, each individual patient chart was manually reviewed by the abstractors to verify the diagnosis based on the clinical laboratory data and bone marrow pathology reports. The initial cohort of patients identified using JDAT included 418 patients. After manual chart review and abstraction, 414 patients were included in the analysis. Hence, the proportion of patients excluded from the analysis was 0.99%. Thus, the final cohort included in the analysis with 100% sensitivity and specificity identified patients with NDMM requiring treatment. We excluded patients with smoldering myeloma, solitary plasmacytoma without systemic therapy, or those treated exclusively outside our institutional network.

### 2.3. Data Collection and Variables

Two co-investigators performed data abstraction with pre-specified rules for abstraction. Each individual patient chart was reviewed manually, diagnosis of newly diagnosed myeloma was confirmed and verified based on the bone marrow report for every patient, and laboratory data were manually abstracted. Notably, this is a single institutional study from a large, single academic health network, encompassing the main academic campus and multiple integrated satellite sites in multiple rural locations. All these locations have unified and integrated laboratory, genetics, radiology, and pathology services. Hence, all laboratory values are available with the same reference ranges and laboratory values. This large academic healthcare network comprises multiple community-based rural satellite sites that are completely integrated within the health system network. These satellites are all rural. The patients receive care near their home wherever they are diagnosed. At Yale Cancer Center/Yale New Haven Health Network, there are 16 satellite locations in rural areas. These centers are staffed by community oncologists with some degree of sub-specialization, who are integrated as faculty members within the Cancer Center. They have an integrated system of chemotherapy pharmacy services; therefore, the delivery of chemoimmunotherapy is similar. From the EHR, we abstracted demographics (age at diagnosis, sex), practice setting at treatment initiation (academic myeloma center vs. community sites), ECOG performance status, Revised International Staging System (R-ISS) stage, immunoglobulin isotype, high-risk cytogenetic status as per the institutional convention (including, but not limited to, del(17p), t(4;14), t(14;16)), bone marrow plasma cell percentage, and key laboratory values at diagnosis (hemoglobin, serum creatinine, and corrected calcium). Transplant eligibility was recorded when explicitly documented or inferred from treatment planning notes. The induction regimen was classified as DARA-based (e.g., triplets/quadruplets with anti-CD38) versus non-DARA-based triplets/quadruplets.

### 2.4. Outcomes

The primary utilization outcome was receipt of a DARA-based front-line regimen. The clinical effectiveness outcome was depth of response at approximately three months after treatment initiation, categorized using the International Myeloma Working Group (IMWG) criteria (complete response, very good partial response, partial response, stable disease, and progression). In this large academic health network, there is unified integrated chemotherapy pharmacy and chemotherapy order templates, which are uniformly applied during treatment. All NDMM induction regimens at our institution consist of 21-day or 28-day cycles. Hence, a 3-month period from the initiation of treatment captures the timepoint immediately following at least three, or up to four cycles of induction. Generally, it is accepted and meaningful to assess the response to treatment in NDMM patients at this timepoint. Hence, we chose a 3-month timepoint for initial response assessment for this cohort. Response timing windows were anchored to the first dose date with a ±2-week window to account for scheduling variability in routine care. A secondary outcome was practice setting variation in adoption, operationalized as the proportion of patients starting induction with DARA at the academic myeloma center versus community sites. Our study was designed as a retrospective, descriptive evaluation of real-world treatment utilization patterns, rather than as a hypothesis-driven comparative study powered to detect differences between subgroups such as academic versus community settings. Accordingly, a formal a priori power calculation for subgroup analyses was not performed.

### 2.5. Statistical Analysis

We summarized continuous variables using medians and interquartile ranges and categorical variables as counts and percentages. Between-group differences were assessed using Pearson’s χ^2^ tests for categorical variables. Primary comparisons contrasted DARA-based versus non-DARA-based induction and evaluated practice setting differences in adoption. At the time of data collection for this cohort, for patients diagnosed between 2020 and 2023, none of the patients had received a front-line isatuximab-based induction regimen. Hence, we are focusing on daratumumab-based combination regimens. The analyses were conducted using standard statistical software (e.g., GraphPad Prism Version 10).

## 3. Results

### 3.1. Cohort

During the study period, we identified 414 patients with NDMM who initiated front-line therapy across the network (median age, 69 years; IQR, 62–77). Most were male (60.6%, n = 251), white (69.1%, n = 286), and non-Hispanic (89.6%, n = 371). At diagnosis, 52.2% (n = 216) had R-ISS stage II, and performance status was generally preserved, with 69.3% (n = 287) having ECOG 0–1. The documented bone marrow plasma cell percentage was evaluable in nearly all patients, at 95.2%. As noted in Table 1, the bone marrow plasma cell percentage was unknown in 4.8% of patients. Care settings were heterogeneous: 61.8% (n = 256) were treated at community practices and 54.1% (n = 224) had commercial insurance. The predominant immunoglobulin isotype was IgG (52.9%, n = 219). Unfavorable-risk cytogenetics were present in 41.3% (n = 171), defined as del(17p), t(4;14), t(14;16), t(14;20), gain or amplification of 1q, 1p deletion, or chromosome 1 translocations. Baseline characteristics are summarized in Table 1.

### 3.2. Front-Line Regimen Utilization and Factors Associated with DARA Receipt

**Figure 1:**
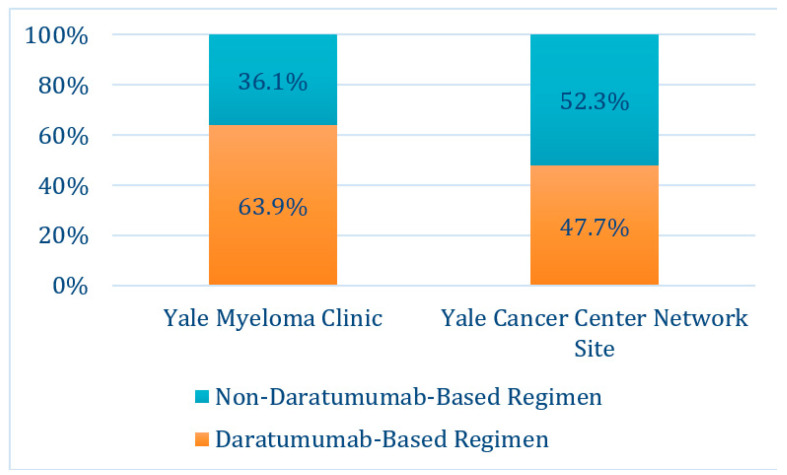
Front-line DARA adoption by practice setting.

The adoption of DARA-based induction was incomplete overall (53.9%, n = 223) and differed by setting (higher at the academic myeloma center than community sites (63.9% vs. 47.7%; *p* = 0.001; Figure 1)). Daratumumab-based regimens included DARA–VRD or DARA–RD. Non-daratumumab regimens included VRD, RD, and rarely VCD (bortezomib–cyclophosphamide, dexamethasone). In unadjusted comparisons, DARA use was more common among patients with ECOG 0–1 (75.3% vs. 62.3%; *p* = 0.009), commercial insurance (60.5% vs. 46.6%; *p* = 0.036), and those considered transplant-eligible (68.2% vs. 55.0%; *p* = 0.006). Differences were also seen based on R-ISS stage (I–II vs. III/unknown, *p* < 0.001), immunoglobulin isotype (*p* = 0.009), serum creatinine (<1.3 vs. ≥1.3 mg/dL, *p* = 0.046), bone marrow plasma cell involvement (≥60% vs. lower, *p* = 0.003), and cytogenetic risk (unfavorable vs. standard, *p* < 0.001). No significant between-group differences were observed for age (*p* = 0.425), gender (*p* = 0.170), race (*p* = 0.556), ethnicity (*p* = 0.989), hemoglobin (*p* = 0.195), calcium (*p* = 0.897), or presence of bone lesions (*p* = 0.996). Notably, the IMWG response was evaluable in all 414 patients evaluated in this cohort. The missingness was noted in terms of R-ISS staging. Namely, as noted in Table 1, the R-ISS stage was unavailable/unknown in 14.5% of patients. The cytogenetics data were missing/unknown in 7.7% of the patients. All patients included in the cohort had received at least three cycles of induction therapy for NDMM.

### 3.3. Early Clinical Response

At approximately three months after treatment initiation, patients who received DARA-based induction achieved deeper responses than those treated without DARA. The distribution of IMWG responses favored the DARA group, with a higher proportion attaining VGPR or better (65.0% [n = 145] vs. 51.3% [n = 98]).

**Figure 2:**
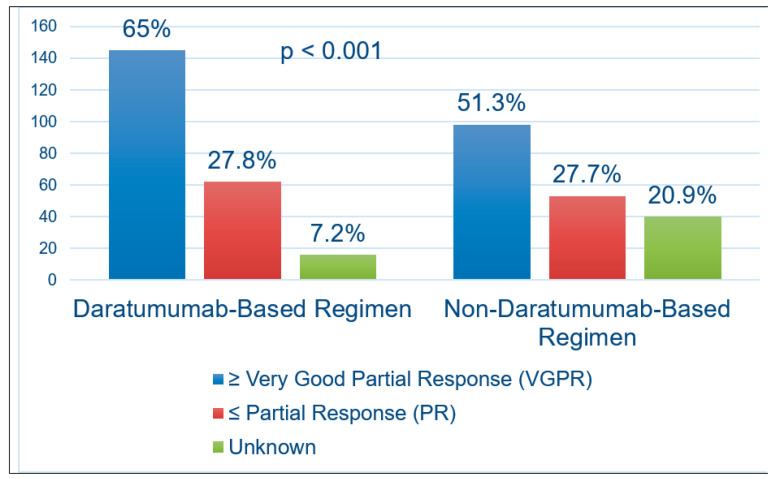
Depth of response at ~3 months by regimen. Distribution of IMWG responses (CR/VGPR/PR/SD/PD) comparing DARA-based vs. non-DARA-based induction.

While longer follow-up is needed to assess progression-free and overall survival, these early response patterns align with the randomized trial data of anti-CD38-based quadruplets (Figure 2).

### 3.4. Temporal Trends

**Figure 3:**
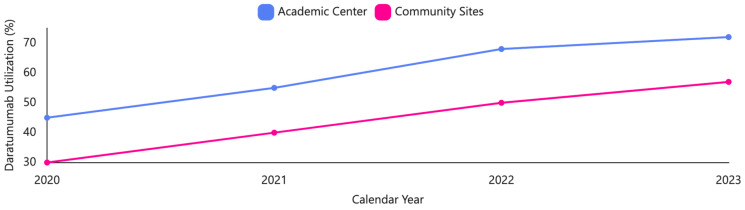
Temporal Adoption of Daratumumab by Practice Setting.

Temporal analysis demonstrated a progressive increase in daratumumab use over the study period, with earlier and more rapid adoption at the academic center compared with at community practices (Figure 3).

## 4. Discussion

Despite the high volume of new myeloma data, particularly with mAbs-based quadruplet therapies, translation into routine oncology practice is often delayed or uneven. This occurrence is multifactorial and reflects how clinicians, systems, and evidence interact in real life. Many myeloma trials contain strict eligibility criteria and have highly selective patient populations, which limits clinicians’ confidence in their applicability to the real-world patient population. Hence, oncologists often hesitate to extrapolate trial results to patients who may look somewhat different from those studied in trials, particularly in front-line settings. Other times, clinicians may delay the adoption of study findings while waiting for the consensus and guideline incorporation. Clinicians may view the results as interesting but premature, rather than immediately practice-changing. Furthermore, even efficacious regimens may not be readily adopted if they are associated with a higher toxicity rate from cytopenias and infections. Additionally, cost, reimbursement, and access represent additional barriers.

In this large, regional, multi-site study, the real-world uptake of DARA-based induction for NDMM has remained heterogeneous and incomplete (53.9%) several years into the modern anti-CD38 era. The strongest signals for DARA use were care at an academic myeloma center and transplant eligibility—both proxies for systematic access to quadruplet regimens and a clinical preference for intensified induction among fitter patients. These observations mirror implementation gradients seen whenever complex regimens transition from trial to practice, where operational constraints, payer processes, and care team comfort can slow diffusion.

The association between DARA use and deeper three-month responses in our cohort parallels trial-level evidence and suggests that, even outside controlled settings, early depth-of-response advantages can be realized. However, because early response does not perfectly predict long-term outcomes for every patient, our findings should be interpreted as supportive of early efficacy signals rather than definitive of survival benefits in this dataset. Follow-up analyses incorporating time-to-event endpoints and attrition across induction, consolidation, and maintenance will be informative. Although early depth of response favored daratumumab-containing regimens, longer follow-up, planned in a subsequent study, is required to determine whether these differences translate into durable improvements in progression-free and overall survival. Future analyses examining treatment sequencing—including second-line daratumumab utilization among patients initially treated with non-DARA regimens—will provide additional insight into evolving real-world practice patterns.

Several factors likely contribute to the observed underutilization of DARA-based regimens in community settings. Firstly, payer authorization and financial toxicity considerations may delay or deter the initiation of DARA-based regimens despite eligibility. Secondly, operational complexity—including infusion chair availability, premedication workflows, and monitoring—can be more challenging to scale in smaller sites. Thirdly, variation in clinician familiarity and evolving comfort with quadruplet regimens may influence perceived risk–benefit assessments for older or comorbid patients. Finally, patient preferences—shaped by travel distance, appointment length, and cultural factors—may steer choices away from regimens perceived as more intensive.

The network context creates a unique opportunity to standardize and support evidence-based care. Embedding a myeloma induction pathway in the EHR that promotes appropriate, guideline-concordant DARA-based options, creates streamlined prior authorization playbooks, and coordinates infusion resources can reduce logistic friction. Structured communication channels between community oncologists and myeloma subspecialists—such as tumor boards—may increase confidence and accelerate adoption when indicated. Community-facing education and shared decision-making tools can help patients understand expected benefits and logistics, thereby improving acceptance and adherence.

### Limitations

This retrospective study relies on routinely collected EHR data and is therefore susceptible to selection bias, documentation variability, and misclassification—particularly for variables not consistently charted (e.g., ECOG, explicit transplant eligibility). Analyses were primarily descriptive, with categorical comparisons using Pearson’s χ^2^ tests; causal inference is limited, and residual confounding is possible. Response assessment at approximately three months may not capture late deepening of the response or early dropouts, and the absence of systematic minimal residual disease testing constrains the interpretation of complete response rates. This cohort represents patients treated from 2020 to 2023 and may not reflect the current utilization of monoclonal antibodies in the front-line treatment of MM. A follow-up study with an updated cohort describing more recent treatment patterns is warranted. Finally, findings from a single regional network may not generalize to other systems with different payer mixes, infusion capacities, or referral patterns.

## 5. Conclusions

Several real-world studies on multiple myeloma demonstrate a time lag of several years between landmark trial publication and prevalent clinical adoption of novel regimens, with delayed acceptance most pronounced in community practice, older patients, and transplant-ineligible populations.

Within the large academic–community network, DARA-based front-line regimens for NDMM were underutilized overall and notably less common at community sites. Receipt of DARA was dependent on transplant eligibility, practice setting, and disease features, and DARA use correlated with deeper early responses at three months. Coordinated implementation strategies at the system, provider, and community levels are well-positioned to narrow adoption gaps and translate trial-proven benefits into routine practice.

## Figures and Tables

**Table 1:** Baseline characteristics of the NDMM cohort (N = 414).

Characteristic	Total (n = 414)	Daratumumab Front-Line (n = 223)	Non-Daratumumab Front-Line (n = 191)	*p*-Value
Age 19–64	137 (33.1)	79 (35.4)	58 (30.4)	0.425
Age 65–75	152 (36.7)	82 (36.8)	70 (36.6)	
Age 76–94	125 (30.2)	62 (27.8)	63 (33.0)	
Male	251 (60.6)	142 (63.7)	109 (57.1)	0.170
Female	163 (39.4)	81 (36.3)	82 (42.9)	
White	286 (69.1)	153 (68.6)	133 (69.6)	0.556
Black	75 (18.1)	38 (17.0)	37 (19.4)	
Asian or Pacific Islander	11 (2.7)	8 (3.6)	3 (1.6)	
Other/Unknown	42 (10.1)	24 (10.8)	18 (9.4)	
Hispanic	34 (8.2)	18 (8.1)	16 (8.4)	0.989
Non-Hispanic	371 (89.6)	200 (89.7)	171 (89.5)	
Unknown Ethnicity	9 (2.2)	5 (2.2)	4 (2.1)	
ECOG 0–1	287 (69.3)	168 (75.3)	119 (62.3)	0.009
ECOG 2–4	108 (26.1)	49 (22.0)	59 (30.9)	
ECOG Unknown	19 (4.6)	6 (2.7)	13 (6.8)	
Medicare	151 (36.5)	68 (30.5)	83 (43.5)	0.036
Medicaid	27 (6.5)	14 (6.3)	13 (6.8)	
Commercial	224 (54.1)	135 (60.5)	89 (46.6)	
Unknown/Uninsured	12 (2.9)	6 (2.7)	6 (3.1)	
Yale Myeloma Clinic	158 (38.2)	101 (45.3)	57 (29.8)	0.001
Yale Cancer Network	256 (61.8)	122 (54.7)	134 (70.2)	
Transplant-Eligible	257 (62.1)	152 (68.2)	105 (55.0)	0.006
Transplant-Ineligible	157 (37.9)	71 (31.8)	86 (45.0)	
Characteristics	Total (n = 414)	DARA-based Regimen Front-Line (n = 223)	Non-DARA-based Regimen Front-Line (n = 191)	*p*-value
Revised International Staging System (R-ISS) Stage I	69 (16.7)	51 (22.9)	18 (9.4)	<0.001
R-ISS Stage II	216 (52.2)	126 (56.5)	90 (47.1)	
R-ISS Stage III	69 (16.7)	28 (12.6)	41 (21.5)	
R-ISS Unknown	60 (14.5)	18 (8.1)	42 (22.0)	
Immunoglobulin Isotype—IgG	219 (52.9)	109 (48.9)	110 (57.6)	0.009
Immunoglobulin Isotype—IgA	95 (22.9)	45 (20.2)	50 (26.2)	
Immunoglobulin Isotype—Light Chain	92 (22.2)	63 (28.3)	29 (15.2)	
Immunoglobulin Isotype—Other (IgD/IgM/non-secretory)	9 (2.2)	6 (2.7)	3 (1.6)	
Hemoglobin < 10 g/dL	209 (50.5)	106 (47.5)	103 (53.9)	0.195
Hemoglobin ≥ 10 g/dL	205 (49.5)	117 (52.5)	88 (46.1)	
Creatinine < 1.3 mg/dL	232 (56.0)	135 (60.5)	97 (50.8)	0.046
Creatinine ≥ 1.3 mg/dL	182 (44.0)	88 (39.5)	94 (49.2)	
Calcium ≤ 11 mg/dL	350 (84.5)	189 (84.8)	161 (84.3)	0.897
Calcium > 11 mg/dL	64 (15.5)	34 (15.2)	30 (15.7)	
Presence of Bone Lesion	271 (65.5)	146 (65.5)	125 (65.4)	0.996
Absence of Bone Lesion	143 (34.5)	77 (34.5)	66 (34.6)	
Bone Marrow Plasma Cell Involvement < 10%	16 (3.9)	7 (3.1)	9 (4.7)	0.003
Bone Marrow Plasma Cell Involvement 10–59%	160 (38.6)	87 (39.0)	73 (38.2)	
Bone Marrow Plasma Cell Involvement ≥ 60%	218 (52.7)	126 (56.5)	92 (48.2)	
Bone Marrow Plasma Cell Involvement Unknown	20 (4.8)	3 (1.3)	17 (8.9)	
Cytogenetic Abnormalities—Standard Risk	211 (51.0)	116 (52.0)	95 (49.7)	<0.001
Cytogenetic Abnormalities—Unfavorable Risk	171 (41.3)	100 (44.8)	71 (37.2)	
Cytogenetic Abnormalities—Unknown	32 (7.7)	7 (3.1)	25 (13.1)	

## Data Availability

Data supporting the reported results are available upon request. Please contact the corresponding author at natalia.neparidze@yale.edu.
